# Development and Validation of Acupuncture Fear Scale

**DOI:** 10.1155/2013/109704

**Published:** 2013-04-03

**Authors:** Ho-Sun Kim, Yun-Ji Kim, Hwa-Jin Lee, Song-Yi Kim, Hyangsook Lee, Dong-Seon Chang, Hyejung Lee, Hi-Joon Park, Younbyoung Chae

**Affiliations:** ^1^Acupuncture and Meridian Science Research Center, College of Korean Medicine, Kyung Hee University, 1 Hoegi-dong, Dongdaemun-gu, Seoul 130-701, Republic of Korea; ^2^Department of Human Perception, Cognition and Action, Max Planck Institute for Biological Cybernetics, 72076 Tübingen, Germany

## Abstract

*Objectives*. Strong aversions to acupuncture have been an obstacle to understanding its intrinsic action of acupuncture. Thus, it is necessary to evaluate the nature and extent of fear of acupuncture treatment. Our study aims to develop and validate an instrument that evaluates a patient's fear of acupuncture treatment. *Methods*. We have developed an acupuncture fear scale, a 16-item instrument which assesses the acupuncture fear score and uses it to survey 275 participants in South Korea, thus testing the reliability and validity of the instrument. *Results*. Internal consistency was high (Cronbach's alpha = 0.935). Test-retest reliability (Spearman's rank correlation coefficient) among 33 participants out of 275 ranged from 0.565 to 0.797 (*P* < 0.001). Principal component analysis revealed two factors accounting for 68% of the variance, which are painful sensation and possible adverse events, respectively. The acupuncture fear scale was positively correlated with the total of fear of pain questionnaire-III (*r* = 0.423, *P* < 0.001). *Conclusions*. The acupuncture fear scale can be a valid and reliable instrument that can measure fear of acupuncture treatment. These results strongly suggest that it would be a clinically useful tool to assess fear of acupuncture in the acupuncture clinic setting and an important instrument to understand the complex social-behavioral component of acupuncture modality.

## 1. Introduction

Fear is an important factor for understanding the characteristics of pain. Pain-related fear influences the perception of the intensity of pain, and it has been reported that fear can be more disabling than pain itself by motivating patients to avoid treatment [1–3]. Fear of injections, for example, is a common concern among patients in healthcare setting. At least 10% of population in a medical setting reports an excessive fear of needles that causes significant avoidance, distress, and/or impairment [4]. Since a person with needle phobia will typically avoid medical care, this condition has been considered a significant impediment in the health care system. In order to minimize needle phobia-related problems and encourage patients to seek treatment, the evaluation of the specific component of fear is very important and has to be considered for any kind of treatment of pain.

Acupuncture is widely used for the treatment of pain, and many clinics, hospitals, and doctors apply it for therapeutic purposes of pain relief. However, there are still a great number of patients hesitant to receive acupuncture treatment, even though it is well known that acupuncture is a safe therapy in clinical practice when general precautions are followed [5]. This fear of receiving acupuncture treatment could be due to the fear of acupuncture needle penetration—a form of instinctive needle phobia—as acupuncture is generally defined as the procedure of inserting and manipulating needles to various specified points on the body surface [6, 7]. Or it could be simply due to the fear of pain, since fine needles penetrating the skin can also evoke a certain degree of pain during the treatment [8]. Another possibility could be also the concern about adverse effects. It is reported that the most commonly feared adverse effects are needle pain (1% to 45%) from treatments, tiredness (2% to 41%), and bleeding (0.03% to 38%) [9]. Regardless of which component is contributing more significantly, evaluating the patients' fear of acupuncture is necessary in order to provide better treatments. 

Several questionnaires have been developed in order to evaluate the specific component of fear. This includes the fear of pain questionnaire-III (FPQ-III), consisting of fear of “severe,” “minor,” and “medical” pain, which has been developed to assess an individual's fear of a variety of different stimuli that may induce pain [10]. “Medical” pain, one of the subscales of FPQ-III, also includes a number of items that represent painful stimuli in a medical setting. The fear of dental pain questionnaire has been developed to understand the role of fear and pain in the context of dental treatment and a patient's well-being [11]. However, to our knowledge, an instrument or questionnaire which assesses fear of acupuncture treatment has not been developed yet. 

The development of Acupuncture Fear Scale would be useful to deal with the fear of acupuncture-induced pain and to minimize fear-induced adverse events in a clinical setting. The purpose of this study was to develop an instrument which assessed the acupuncture fear and then conduct a survey to test the reliability and validity of the instrument. We obtained reliable information regarding the structure of the response patterns of the Acupuncture Fear Scale. We also tested construct validity by correlating the score of Acupuncture Fear Scale to the ratings of FPQ-III. 

## 2. Methods

### 2.1. Development of the Acupuncture Fear Scale Questionnaire

We used a structured interview in which responses are open-ended to a set of structured questions. This format was useful in reducing the interviewer's bias and obtaining expansive information from the interviewees. The interviewees (*N* = 50) were asked to describe what they felt afraid of when treated with acupuncture. The answers were roughly categorized into two issues: sensation of pain and possible adverse events. Based on this tendency and qualitative descriptions, four experts—two in the field of acupuncture and one for each Korean language and cognitive psychology—were asked to create the items which describe acupuncture treatment-related situations that may induce fear. The contents of all items were judged for clarity, difficulty, and relevance among them, resulting in a list of 16 items (Table 1). All the experts reviewed the expressions of the 16 items from differing points of view. Five-point Likert-type scales from 1 (not at all) to 5 (extremely) were used. The Korean version of the acupuncture fear scale was translated into English using standardized forward and backward translation procedures [12].

### 2.2. Participants

A total of 275 participants (male = 74, female = 201) were recruited, and all subjects were between 14 and 66 years old (mean age = 35.15, SD = 12.87). Exclusion criteria were any kind of cognitive problems. People who were working or majoring in the medical field were excluded as well. Thirty-three test-retest participants were randomly chosen out of these 275 questionnaire participants. They received a detailed explanation of the questionnaire, and written consent was obtained. Basic demographic data such as age and gender were acquired from all participants by the questionnaire. This investigation was conducted in accordance with the guidelines of the human subjects committee of Kyung Hee University, Seoul, Republic of Korea. 

### 2.3. Procedures

All participants were asked to fill out the Acupuncture Fear Scale questionnaire on their own. All participants completed the questionnaire in less than 5 minutes. The test-retest data of 33 random subjects were obtained in the same manner five weeks later. 

In order to test the validity of the Acupuncture Fear Scale, all participants also filled out the questions on the FPQ-III which is a 30-item self-report questionnaire assessing an individual's fear of a variety of stimuli that may produce pain. The FPQ-III includes three subscales, severe pain, minor pain, and medical pain, and it has been reported to provide evidence for the validity and utility of the FPQ-III [10].

### 2.4. Statistical Analysis

We evaluated the internal consistency of the Acupuncture Fear Scale by computing item-total correlations and Cronbach's  *α*. Spearman's rank correlation coefficients were calculated to determine test-retest reliability. The paired sample  *t*-test was also implemented to complement the correlation coefficient. 

To test construct validity, we performed a principal component analysis to explore the potential number and characteristics of the domains. Varimax rotation was performed to more specific response patterns present, with an eigen value greater than one as the criterion. We also evaluated the construct validity by correlating scores on the Acupuncture Fear Scale and FPQ-III. Data analyses were performed using the Statistical Package for Social Sciences for Windows 18.0 (SPSS, Chicago, IL, USA). Statistical significance level was set for *P* < 0.05.

## 3. Results

### 3.1. Sample Characteristics

A total of 275 subjects (mean age: 35.15 ± 12.87) participated in this survey (Table 2) where seventy-three percent were females. One hundred and three participants (*N* = 103, 37.5%) had no previous experience of acupuncture treatment, and one hundred and seventy-two participants (*N* = 172, 62.5%) had previous experience of acupuncture treatments within the past five years. Sixty-eight participants (*N* = 68, 24.7%) had pain-related conditions.

### 3.2. Properties of the Scale

The response patterns to the 16-item Acupuncture Fear Scale of all 275 participants are shown in Table 3. The score of the final instrument ranged from 16 to 68 of a possibility from 16 to 80. The mean was 36.63, with a standard deviation of 11.57, and a median of 36.

### 3.3. Internal Consistency

Cronbach's  *α*  was 0.935 for the mean of the item total. It did not become greater when any of the 16 items were removed. Each item had an item-total correlation value greater than 0.5, and five items (AFS4, 5, 7, 8, and 10) had correlation values greater than 0.7 (Table 4).

### 3.4. Test-Retest Reliability

There was no significant difference in the first test and the second test of any item in the Acupuncture Fear Scale. The retest was conducted 5 weeks after the first test. All items were not significantly different at *P* < 0.05 (Table 5). All items exhibited high Spearman's rank correlation coefficients ranged from 0.565 to 0.797.

### 3.5. Factor Analysis

The 16 items were subjected to factor analysis to examine the factorial validity of the Acupuncture Fear Scale using principal component extraction and varimax rotation with an Eigen value over 1.0 as the criterion. The two-factor solution explained an acceptable amount of variance (67.741%) in the response (Table 6). Factor 1 (entitled as “sensation of pain”) was made up of 8 items with factor loadings of 0.675 to 0.873, which accounted for 51.296% of variance. Factor 2 (entitled as “possible adverse events”) consisted of the remaining 8 items with factor loadings of 0.652 to 0.846, which accounted for 16.445% of the variance. When we evaluated the internal consistency of the two factors, Cronbach's  *α*  was 0.939 and 0.915 for the two factor scores, respectively.

### 3.6. Validity

To test construct validity, a correlation analysis was conducted between the Acupuncture Fear Scale and FPQ-III questionnaire (Table 7). The total of Acupuncture Fear Scale was positively correlated with the total of FPQ-III (*r* = 0.423, *P* < 0.001). Factor 1 of the Acupuncture Fear Scale (*r* = 0.413, *P* < 0.001), that is, sensation of pain, exhibited greater correlations with the total of FPQ-III than factor 2 of the Acupuncture Fear Scale (*r* = 0.326, *P* < 0.001), that is, adverse events. In addition, the Acupuncture Fear Scale showed highest correlations with the subscale “fear of Medical pain” among the three subscales of the FPQ-III (*r* = 0.498, *P* < 0.001). 

### 3.7. Subgroup Analysis

We also looked at the influence of factors such as gender, acupuncture experience, and diseases with pain symptom and compared answers to the Acupuncture Fear Scale based on those factors. Female participants scored higher on the Acupuncture Fear Scale compared to male participants (38.14 ± 11.69 versus 32.51 ± 10.23,  *t* = 3.656, *P* < 0.001). Participants without acupuncture experience also showed higher scores on the Acupuncture Fear Scale compared to those with acupuncture experience (40.60 ± 11.15 versus 34.53 ± 10.79,  *t* = 5.262, *P* < 0.001). Younger participants (age < 40) exhibited higher scores on the Acupuncture Fear Scale compared to older participants (age > 40) (37.81 ± 11.87 versus 34.52 ± 10.40,  *t* = 2.292, *P* < 0.001). There were no significant differences between normal participants and patients with pain (35.52 ± 11.42 versus 35.53 ± 10.47,  *t* = 0.626, *P* > 0.532).

## 4. Discussion

Fear can be a major impediment to apply necessary health care measures, and it is important that reliable and valid assessment tools are available to measure the cause and extent of a fear [2]. Several questionnaires have been developed in the past, such as the fear of dental pain questionnaire [11] or the fear of pain questionnaire [10]. However, there has not been an instrument which assessed the fear of acupuncture treatment, even though acupuncture became increasingly important and popular for the treatment of pain in the past years [5]. The present study was designed to systematically develop an instrument which assesses fears of acupuncture and also test the reliability and validity of this instrument. 

To test its reliability, we evaluated internal consistency and test-retest reliability in this study. Cronbach's  *α*  is believed to indirectly indicate the degree to which a set of items or variables measure a single unidimensional latent construct. It generally requires a reliability of 0.70 or higher. In the present study, Cronbach's  *α*  coefficient for the Acupuncture Fear Scale reveals strong internal consistency, with a value of 0.935, indicating a reliable measurement of Acupuncture Fear Scale. Furthermore, Spearman's rank correlation coefficient ranged from 0.565 to 0.797 (*P* < 0.001), suggesting that all items of the Acupuncture Fear Scale have a good test-retest reliability. As all the items of the Acupuncture Fear Scale revealed good internal consistency and test-retest reliability, we can conclude that it is a reliable instrument with a good repeatability and reproducibility. 

For the validity of the proposed Acupuncture Fear Scale, we evaluated a correlation analysis between the Acupuncture Fear Scale and the FPQ-III questionnaire. In the current study, we demonstrated that the total of the Acupuncture Fear Scale revealed a positive correlation with the total of the FPQ-III (*r* = 0.423) and the highest correlation with the subscale fear of “medical” pain among three subscales of the FPQ-III (*r* = 0.498). Our results were consistent with the previous finding that fear of dental pain questionnaire exhibited the highest correlation with the subscale fear of “medical” pain of the FPQ-III [11]. It is speculated that items of the Acupuncture Fear Scale contain fear of pain associated with the general medical settings. We also found that the subscale of acupuncture fear, the sensation of pain subscale (*r* = 0.413), exhibited greater correlations with the total of the FPQ-III than the other subscale of acupuncture fear, adverse events subscale (*r* = 0.326). It might be deduced that the sensation of pain subscale could more directly represent the degree of fear of pain than the possible adverse event subscale. 

When we asked participants to describe what they were afraid of regarding acupuncture treatment in our preliminary study, most of the answers were narrowed down to two categories: sensation of pain and possible adverse events. In order to examine the factorial validity of the acupuncture fear scale, we conducted a principal component analysis, which revealed two factors accounting for 67.7% of the variance. Factor 1 is made up of 8 items related with sensation of pain, which explained 51.3% of variance. Factor 2 consists of 8 items associated with possible adverse events, which explained 16.4% of variance. These results indicate that most of the participants were afraid of being treated with acupuncture because of the inevitable sensation of pain and possible adverse events. This has been also taken into account by the two subscales of the acupuncture fear scale in the present study, including these two factors: sensation of pain and possible adverse events.

The results of this study lead to further implications. As acupuncture stimulation can give rise to feelings of faintness upon exposure to feared stimuli, for example, vasovagal syncope in needle-phobic patients, fear of acupuncture pain should be carefully managed in an acupuncture clinic [13]. It is reported that an acupuncture-induced sensation of pain and physiological responses were altered by cognitive manipulation regarding acupuncture modalities [7]. Thus, fear of acupuncture-evoked pain might be able to be alleviated through cognitive therapy and other methods [14]. Regarding the fear of possible adverse events, it might be necessary to provide people with exact health information of adverse events regarding acupuncture treatment, in order to correct exaggerated aversion to acupuncture. There are studies showing that acupuncture is well known to be a safe therapy with a low risk of adverse events in clinical practice [5]. Understanding the patients' fear of acupuncture treatment in a clinical setting would be also useful to minimize fear-induced adverse events and to build a more desirable doctor-patient relationship. 

 There are a few limitations which need to be considered in this study. As the Acupuncture Fear Scale was originally developed in Korean and translated into English, the English version of this instrument needs to be further tested in more diverse populations. In addition, because of the higher cultural familiarity and acceptance of acupuncture in East Asian countries, the results might differ in western countries, and therefore, the Acupuncture Fear Scale should be also validated in western countries. As the high value of Cronbach's alpha (>0.90) may suggest unnecessary duplication of content across items [15], it is necessary to develop a short version of the Acupuncture Fear Scale in the future. Since the present study included varying population samples, these various characteristics of populations could be possible confounding factors. In the subgroup analysis, we found that the Acupuncture Fear Scale varied significantly by their gender, age, and experience of acupuncture treatment. In order to quantify the prevalence of a patient's fear of acupuncture score, it is required to compare different populations and subpopulations or to track population shifts over time with a larger number of populations in the future study. It is recommended that the Acupuncture Fear Scale is used to screen all the acupuncture treatment patients for fear of acupuncture to enable a more tailored and effective acupuncture treatment experience. 

In sum, all the items of the Acupuncture Fear Scale revealed good internal consistency and test-retest reliability. Principal component analysis revealed two factors, such as sensation of pain and possible adverse events. The Acupuncture Fear Scale has proven to be well validated. Therefore, we can conclude that the Acupuncture Fear Scale we have developed in the current study is a valid and reliable instrument to assess fear of acupuncture treatment. Moreover, we suggest that this questionnaire would be a clinically useful tool to prevent possible adverse events of acupuncture and an important instrument to understand the complex social-behavioral components of the acupuncture modality. 

## Figures and Tables

**Table 1:** The 16 items of the acupuncture fear scale.

Acupuncture fear scale items	
AFS1. Seeing an acupuncture needle	
AFS2. Seeing an acupuncture needle puncturing my skin	
AFS3. Seeing an acupuncture needle puncturing another person's skin	
AFS4. The stinging sensation of an acupuncture needle puncturing my skin	
AFS5. An acupuncture needle entering my body's flesh	
AFS6. Acupuncture treatment on my face	
AFS7. Acupuncture treatment on my hands and/or feet	
AFS8. Acupuncture treatment on my torso	
AFS9. The possibility of having acupuncture needles in the wrong areas	
AFS10. The possible occurrence of unpleasant sensation due to acupuncture treatment	
AFS11. The possible occurrence of nerve damage due to acupuncture treatment	
AFS12. Persistent pain possibly occurring after acupuncture treatment	
AFS13. The possible occurrence of bleeding due to acupuncture treatment	
AFS14. The possibility of bruising due to acupuncture treatment	
AFS15. The possibility of an infection occurring due to acupuncture treatment	
AFS16. The possibility of damage to other parts of my body (parts of the body not exposed to acupuncture treatment) due to acupuncture treatment	

**Table 2:** Demographics of the study's participants (*N* = 275).

	Number (%)
Gender	
Women	201 (73.09)
Men	74 (26.91)
Prior acupuncture experience (within the last 5 years)	
Never	103 (37.45)
1–3 times	79 (28.73)
4–6 times	26 (9.45)
7–9 times	9 (3.27)
≥10 times	45 (16.36)
Pain-related conditions	
Yes	68 (24.73)
No	194 (70.55)

*Total sample 275, within cells totals may not sum to 275 because of missing data.

**Table 3:** The response patterns to the acupuncture fear scale (*N* = 275).

Item	Not at all (1)	A little (2)	Fair amount (3)	Very much (4)	Extremely (5)
AFS1	82 (29.8)	111 (40.4)	58 (21.1)	20 (7.3)	3 (1.1)
AFS2	73 (26.5)	105 (38.2)	57 (20.7)	31 (11.3)	7 (2.5)
AFS3	115 (41.8)	85 (30.9)	50 (18.2)	22 (8.0)	3 (1.1)
AFS4	67 (24.4)	116 (42.2)	51 (18.5)	38 (13.8)	3 (1.1)
AFS5	71 (25.8)	113 (41.1)	50 (18.2)	36 (13.1)	5 (1.8)
AFS6	14 (5.1)	63 (22.9)	70 (25.5)	87 (31.6)	41 (14.9)
AFS7	82 (29.8)	106 (38.5)	60 (21.8)	23 (8.4)	4 (1.5)
AFS8	75 (27.3)	106 (38.5)	69 (25.1)	24 (8.7)	1 (0.4)
AFS9	26 (9.5)	88 (32.0)	87 (31.6)	62 (22.5)	12 (4.4)
AFS10	67 (24.4)	102 (37.1)	69 (25.1)	31 (11.3)	5 (1.8)
AFS11	47 (17.1)	97 (35.3)	67 (24.4)	47 (17.1)	17 (6.2)
AFS12	78 (28.4)	104 (37.8)	61 (22.2)	26 (9.5)	5 (1.8)
AFS13	102 (37.1)	105 (38.2)	46 (16.7)	21 (7.6)	0 (0)
AFS14	108 (39.3)	117 (42.5)	34 (12.4)	14 (5.1)	2 (0.7)
AFS15	63 (22.9)	92 (33.5)	67 (24.4)	42 (15.3)	11 (4.0)
AFS16	73 (26.5)	108 (39.3)	65 (23.6)	26 (9.5)	3 (1.1)

AFS: acupuncture fear scale, number (percentage).

**Table 4:** Internal consistency of the acupuncture fear scale (*N* = 275).

Item	Score (mean ± SD)	Item-total correlation
AFS1	2.09 ± 0.95	0.686
AFS2	2.25 ± 1.05	0.673
AFS3	1.96 ± 1.01	0.625
AFS4	2.25 ± 1.01	0.715
AFS5	2.24 ± 1.04	0.753
AFS6	3.28 ± 1.13	0.628
AFS7	2.13 ± 0.98	0.742
AFS8	2.16 ± 0.94	0.737
AFS9	2.80 ± 1.03	0.660
AFS10	2.29 ± 1.02	0.735
AFS11	2.60 ± 1.14	0.659
AFS12	2.18 ± 1.01	0.692
AFS13	1.95 ± 0.92	0.637
AFS14	1.85 ± 0.88	0.641
AFS15	2.44 ± 1.12	0.515
AFS16	2.19 ± 0.97	0.560

AFS: acupuncture fear scale, *Cronbach's *α* coefficient of the total is 0.935; each item scored by the patient is on a 5-point Likert scale, from 1 (not at all) to 5 (extremely).

**Table 5:** Test-retest reliability of the acupuncture fear scale (*N* = 33).

Item	Score in the 1st test (mean ± SD)	Score in the 2nd test (mean ± SD)	*P* value*	Spearman's rank coefficient (95% CI)
AFS1	2.12 ± 0.99	2.12 ± 1.08	1.000	0.597 (0.331, 0.688)**
AFS2	2.48 ± 1.09	2.33 ± 1.16	0.344	0.680 (0.471, 0.829)**
AFS3	2.00 ± 1.03	2.18 ± 1.07	0.245	0.649 (0.416, 0.774)**
AFS4	2.42 ± 1.12	2.24 ± 0.90	0.280	0.576 (0.299, 0.656)**
AFS5	2.39 ± 1.05	2.27 ± 0.91	0.441	0.598 (0.332, 0.690)**
AFS6	3.64 ± 1.14	3.42 ± 1.09	0.109	0.781 (0.690, 1.048)**
AFS7	2.33 ± 1.11	2.33 ± 1.11	1.000	0.797 (0.732, 1.090)**
AFS8	2.12 ± 0.93	2.27 ± 0.98	0.169	0.790 (0.714, 1.071)**
AFS9	2.94 ± 1.09	3.03 ± 1.07	0.500	0.750 (0.615, 0.973)**
AFS10	2.24 ± 0.97	2.45 ± 1.12	0.214	0.586 (0.314, 0.672)**
AFS11	2.70 ± 1.26	2.76 ± 1.23	0.690	0.759 (0.636, 0.994)**
AFS12	2.21 ± 1.22	2.30 ± 1.10	0.598	0.648 (0.414, 0.772)**
AFS13	1.97 ± 1.02	1.97 ± 0.92	1.000	0.569 (0.288, 0.646)**
AFS14	2.06 ± 1.06	2.12 ± 0.93	0.712	0.565 (0.282, 0.640)**
AFS15	2.33 ± 1.22	2.36 ± 1.22	0.851	0.716 (0.542, 0.899)**
AFS16	2.27 ± 1.15	2.18 ± 1.04	0.521	0.736 (0.584, 0.942)**

AFS: acupuncture fear scale; *paired sample *t*-test was used; **test-retest reliability is reported with the Spearman's rank correlation coefficient; all items were highly significant at *P* < 0.001.

**Table 6:** Factor variance explained and item factor loadings for the final solution.

Item	Factor loadings	Variance explained (%)
Factor 1: sensation of pain		51.296
AFS1	0.856	
AFS2	0.873	
AFS3	0.764	
AFS4	0.849	
AFS5	0.840	
AFS6	0.675	
AFS7	0.828	
AFS8	0.784	
Factor 2: possible adverse events		16.445
AFS9	0.686	
AFS10	0.710	
AFS11	0.810	
AFS12	0.841	
AFS13	0.735	
AFS14	0.652	
AFS15	0.773	
AFS16	0.846	

AFS: acupuncture fear scale.

**Table 7:** Correlation coefficients between the acupuncture fear scale and the fear of pain questionnaire (*N* = 275).

	FPQ-III total	FPQ-III severe	FPQ-III minor	FPQ-III medical
AFS total	0.423	0.279	0.346	0.498
AFS factor 1	0.413	0.265	0.311	0.519
AFS factor 2	0.326	0.223	0.294	0.349

AFS: acupuncture fear scale; all correlations were significant at *P* < 0.001.

## Appendix

### Acupuncture Fear Scale

Name:

Date:

*Instructions.* The following items describe possible reasons that would make you fear receiving acupuncture treatment. Please look at each item and think about how fearful you are with such reasons. Circle one rating per item to rate your fear of acupuncture treatment in relation to each reason.

1. Seeing an acupuncture needle.
  1. Not at all.
  2. A little.
  3. Fair amount.
  4. Very much.
  5. Extreme.
2. Seeing an acupuncture needle puncturing my skin.
  1. Not at all.
  2. A little.
  3. Fair amount.
  4. Very much.
  5. Extreme.
3. Seeing an acupuncture needle puncturing another person's skin.
  1. Not at all.
  2. A little.
  3. Fair amount.
  4. Very much.
  5. Extreme.
4. The stinging sensation of an acupuncture needle puncturing my skin.
  1. Not at all.
  2. A little.
  3. Fair amount.
  4. Very much.
  5. Extreme.
5. An acupuncture needle entering my body's flesh.
  1. Not at all.
  2. A little.
  3. Fair amount.
  4. Very much.
  5. Extreme.
6. Acupuncture treatment on my face.
  1. Not at all.
  2. A little.
  3. Fair amount.
  4. Very much.
  5. Extreme.
7. Acupuncture treatment on my hands and/or feet.
  1. Not at all.
  2. A little.
  3. Fair amount.
  4. Very much.
  5. Extreme.
8. Acupuncture treatment on my torso.
  1. Not at all.
  2. A little.
  3. Fair amount.
  4. Very much.
  5. Extreme.
9. The possibility of having acupuncture needles in the wrong areas.
  1. Not at all.
  2. A little.
  3. Fair amount.
  4. Very much.
  5. Extreme.
10. The possible occurrence of unpleasant sensation due to acupuncture treatment.
  1. Not at all.
  2. A little.
  3. Fair amount.
  4. Very much.
  5. Extreme.
11. The possible occurrence of nerve damage due to acupuncture treatment.
  1. Not at all.
  2. A little.
  3. Fair amount.
  4. Very much.
  5. Extreme.
12. Persistent pain possibly occurring after acupuncture treatment.
  1. Not at all.
  2. A little.
  3. Fair amount.
  4. Very much.
  5. Extreme.
13. The possible occurrence of bleeding due to acupuncture treatment.
  1. Not at all.
  2. A little.
  3. Fair amount.
  4. Very much.
  5. Extreme.
14. The possibility of bruising due to acupuncture treatment.
  1. Not at all.
  2. A little.
  3. Fair amount.
  4. Very much.
  5. Extreme.
15. The possibility of an infection occurring due to acupuncture treatment.
  1. Not at all.
  2. A little.
  3. Fair amount.
  4. Very much.
  5. Extreme.
16. The possibility of damage to other parts of my body (parts of the body not exposed to acupuncture treatment) due to acupuncture treatment.
  1. Not at all.
  2. A little.
  3. Fair amount.
  4. Very much.
  5. Extreme.
